# Radix Angelica Sinensis and Radix Hedysari Ultrafiltration Extract Protects against X-Irradiation-Induced Cardiac Fibrosis in Rats

**DOI:** 10.1155/2020/4675851

**Published:** 2020-04-21

**Authors:** Chengxu Ma, Xinke Zhao, Juan Chang, Huan Guo, Huiping Wei, Zhaoyuan Fu, Yingdong Li

**Affiliations:** ^1^College of Integrated Traditional Chinese and Western Medicine, Gansu University of Chinese Medicine, Lanzhou 730000, China; ^2^Affiliated Hospital of Gansu University of Chinese Medicine, Lanzhou 730000, China; ^3^The Center of Traditional Medical Diagnosis and Treatment, Gansu Provincial Hospital, Lanzhou 730000, China; ^4^School of Basic Medical Sciences, Lan Zhou University, Lanzhou 730000, China

## Abstract

Radiation-induced myocardial fibrosis (RIMF) is the main pathological change associated with radiation-induced heart toxicity after radiation therapy in patients with thoracic tumors. There is an antifibrosis effect of Radix Angelica Sinensis and Radix Hedysari (RAS-RH) ultrafiltration extract from Danggui Buxue decoction (DBD) in X-irradiation-induced rat myocardial fibrosis, and this study aimed to investigate whether that effect correlated with apoptosis and oxidative stress damage in primary rat cardiac fibroblasts; further, the potential mechanisms were also explored. In this study, we first found that the RAS-RH antifibrosis effect was associated with the upregulation of microRNA-200a and the downregulation of TGF-*β*1/smad3 and COL1*α*. In addition, we also found that the antifibrosis effect of RAS-RH was related to the induction of apoptosis in primary rat cardiac fibroblasts and to the prevention of damage caused by reactive oxygen species (ROS). Interestingly, primary rat cardiac fibroblasts exposed to X-ray radiation underwent apoptosis less frequently in the absence of RAS-RH. Therefore, RAS-RH has the ability to protect against fibrosis, which could be occurring through the induction of apoptosis and the resistance to oxidative stress in rats with X-irradiation-induced myocardial fibrosis; thus, in a model of RIMF, RAS-RH acts against X-irradiation-induced cardiac toxicity.

## 1. Introduction

Radiation-induced heart disease (RIHD) is associated with mortality and morbidity in patients with thoracic malignancies who undergo radiotherapy (RT), and RIHD limits the effect of tumor management. Although typical radiotherapy regimens continue to improve, the treatments still cause cardiac toxicity [1]. In the first two days after RT, early fibrosis occurs in the heart [2]. Cardiac fibroblasts exposed to radiation play a vital role in proliferation, apoptosis, and oxidative damage, and they secrete collagen and extracellular matrix [3]; further, early macrophage-rich plaques form in the intima of the vessel wall after radiation [4]. Radiation can accelerate the development of coronary artery disease and diffuse interstitial fibrosis of the myocardium with endothelial cell effacement [5], which is a typical manifestation of radiation-induced myocardial fibrosis (RIMF).

Many profibrotic molecules and signal cascades are involved in the occurrence of RIMF. The reaction between X-rays and water molecules in cardiac fibroblasts produces reactive oxygen species (ROS) [6]. ROS directly activate TGF-*β*1/smad3, which promotes fibroblast proliferation and eventually diffuse fibrosis in heart tissue. Circulating microRNA-200a may represent a biomarker for diffuse myocardial fibrosis in patients with hypertrophic cardiomyopathy [7], and it may protect against apoptosis by inhibiting ROS in cardiomyocytes [8]. Taken together, these data show that TGF-*β*1/Smad3, ROS, and microRNA-200a are highly correlated with RIMF.

To date, there is no adequate evidence to support the idea that RIMF can be efficiently prevented by treatment with existing drugs, such as ACE inhibitors, amifostine, and statins. Danggui Buxue is an ancient Chinese decoction which is a famous “yiqi-buxue” herbal preparation containing Radix Angelica Sinensis and *Astragalus* membranaceus at a ratio of 1 : 5; it appears to have a more potent protective effect compared to its component herb extracts in myocardial ischemia-reperfusion injury [9], and it has an antifibrosis effect in diabetic nephropathy and liver fibrosis [10, 11]. Radix Hedysari contains most of the chemical constituents that have antihepatic fibrosis effects, and it has a higher quantity of formononetin than Astragali Radix, which has antiapoptosis and antioxidation effects in the rat heart [12, 13]. Therefore, we selected Radix Hedysari instead of Astragali Radix to prepare Radix Angelica Sinensis and Radix Hedysari (RAS-RH) ultrafiltration extract from the Danggui Buxue decoction, and we subsequently confirmed that RAS-RH has a correlative protective effect against RIMF [14]. In this paper, we investigated the possible protective mechanism of ASRH in rats with RIMF.

## 2. Materials and Methods

### 2.1. Animal Experiments

#### 2.1.1. Animal Groups and Treatment

All animal experiments followed guidelines regarding the humane use and care of laboratory animals. Experiments were overseen by the responsible authorities and received approval from the Animal Ethics Committee of Gansu University of Chinese Medicine (Lanzhou, China). Male Wistar rats (11–14 weeks old, 200 to 230 g, the Animal Breeding Center of Gansu University of Chinese Medicine, Lanzhou, China) were randomly divided into three groups after about one week of acclimation in a temperature-controlled room (21°C; relative humidity 50–70%). The first group of rats underwent sham irradiation, and they served as the control group (*n* = 7). The second group of rats was irradiated with a single 8 Gy dose of total body irradiation (TBI) that was administered using a PXi-225 (North Branford, USA) in a shielded facility for 30 days (*n* = 12, X-ray group). The third group of rats was given RAS-RH by intragastric administration (50 mg/kg/day) for 30 days at 24 hours after TBI (*n* = 10, RAS-RH + X-ray group), and RAS-RH was prepared as described previously [14].

#### 2.1.2. Histopathological Analysis

Hematoxylin and eosin (H&E) staining was used to show the inflammatory state of the rat hearts. The entire hearts of rats from the control, X-ray, and RAS-RH + X-ray groups were stored in 10% formalin (*v*/*v*) and then were embedded in paraffin. Then, 4 *μ*m sections were stained with hematoxylin and eosin (H&E) according to standard methods. Images were captured with the light microscope (Olympus BX51, Japan).

#### 2.1.3. Masson's Trichrome Staining Analysis of Collagen Composition

Tissue slides (4 *μ*m) prepared from the paraffin-embedded hearts from the control, X-ray, and RAS-RH + X-ray groups of rats after 1 month of treatment were used for histological analysis. Collagen deposition was determined by Masson's trichrome staining according to standard methods.

#### 2.1.4. Determination of TGF-*β*1, TnT, Brain Natriuretic Peptide (BNP), and ROS Content

TGF-*β*1, TnT, brain natriuretic peptide (BNP), and ROS content were measured with a TGF-*β*1 ELISA kit (R&D Systems, Shanghai, China), a rat TnT ELISA kit, a rat BNP ELISA kit (Meimian, Jiangsu, China), and a rat ROS ELISA kit (Ren Jiebio, Shanghai, China), respectively. Briefly, serum from the control, X-ray, and RAS-RH + X-ray rats was collected and then centrifuged at 4000 rpm for 10 minutes. The supernatant was collected and incubated in plates precoated with purified rat TGF-*β*1, TnT, BNP, and ROS antibodies. The detection limits of the TGF-*β*1, TnT, BNP, and ROS ELISA kits were <4.6 pg/mL, 1 ng/L, 3 ng/L and 1.0 U/mL, respectively.

### 2.2. Cell Experiments

#### 2.2.1. Primary Cardiac Fibroblast Isolation

Primary cardiac fibroblasts were prepared by enzymatic dissociation of heart tissue from the control, X-ray, and RAS-RH + X-ray groups as described in [15]with modifications. Briefly, the hearts were finely minced and subjected to a series of incubations with trypsin (0.5 mg/mL, Solarbio, Beijing, China) in phosphate-buffered saline (PBS, pH 7.4) containing 1 mg/mL collagenase II (Gibco, Beijing, China). Trypsinization was stopped by the addition of 10% serum. After collection by centrifugation, cardiac fibroblasts were isolated by a 30-minute period of preplating in MEM (Solarbio, Beijing, China) supplemented with 5% BCS (Solarbio, Beijing, China) at 37°C in 5% CO_2_, and then they were used for various assays described below.

#### 2.2.2. Detection of Apoptosis

Apoptotic cardiac fibroblasts were evaluated by performing flow cytometric analysis. The cells were washed twice with PBS and then were suspended in 100 *μ*L of binding buffer containing 5 *μ*L of annexin V-fluorescein isothiocyanate (FITC) and 5 *μ*L of propidium iodide (PI) (Multisciences, Zhejiang, China) in the dark at room temperature. After 15 minutes, the cell suspensions were immediately analyzed with a flow cytometer (ACEA Biosciences, Hangzhou, China) using Cellquest software (ACEA Biosciences, Hangzhou, China).

#### 2.2.3. Total RNA Isolation and cDNA Synthesis

Total cellular RNA was extracted using TRIzol reagent (Ambion, Carlsbad, CA). First-strand cDNA was synthesized from microRNA with a microRNA First-Strand cDNA Synthesis kit (Fulen Gene, Guangzhou, China). First-strand cDNA was synthesized from mRNA with a GoScript™ RT reagent kit (Promega, Beijing, China) according to the protocol provided by the manufacturer.

#### 2.2.4. Quantitative Real-Time Polymerase Chain Reaction

The expression level of mature microRNA-200a was measured by real-time PCR with an All-in-One™ qPCR Mix kit (Fulen Gene, Guangzhou, China). The cycling parameters were 95°C for 10 minutes, followed by 95°C (10 s) and 60°C (20 s). The relative abundance of microRNA-200a was calculated with U6 snRNA normalization, and the cycle threshold (Ct) value was used for analysis. The mRNA expression levels of collagen-I (COL1*α*), TGF-*β*1, Smad3, TNF-*α*, caspase-3, and GAPDH (internal control) were analyzed by real-time PCR with a GoTaq® qPCR Master Mix kit (Promega, Beijing, China). PCR mixtures included cDNAs at an appropriate dilution, qPCR Master Mix, and 10 mM primers in a total reaction volume of 10 *μ*l. The expression data (after being normalized to GAPDH levels) were analyzed using the 2^−ΔΔCt^ method. The primers are shown in Table 1.

#### 2.2.5. Western Blotting

Rat cardiac fibroblasts were homogenized and lysed with RIPA lysis buffer (Promega, Beijing, China). Total protein was isolated, and protein content was quantified using a BCA kit (Thermo Fisher Scientific, Waltham, MA). Twenty-five micrograms of protein was separated on SDS gels by SDS-PAGE and then was transferred to a polyvinylidene difluoride (PVDF) membranes. The membranes were then blocked at room temperature for 1 hour in Tris-buffered saline with 0.2% Tween 20 (TBST) containing 5% skim milk. Then, the membranes were incubated overnight at 4°C with rabbit anti-Col1*α* (ab64883, Abcam, Beijing, China), rabbit anti-TGF-*β*1 (GTX45121, Gene Tex, Texas, UK), mouse anti-Caspase-3 (GTX13585, Gene Tex, Texas, UK), rabbit anti-TNF-*α* (GTX26671, Gene Tex, Texas, UK), and rabbit anti-Smad3 (#9523, Cell Signaling Technology, Beijing, China). Finally, membranes were washed three times with TBST and were incubated for 1 hour with horseradish peroxidase-conjugated goat anti-rabbit or goat anti-mouse secondary antibody (ImmunoWay Biotechnology, Jiangsu, China). Protein bands were visualized using enhanced chemiluminescence (Millipore, Zurich, Switzerland).

### 2.3. Data Analysis

The data in graphs are presented as the mean ± standard deviation (SD) unless otherwise stated. The significance of differences between groups was determined by one-way ANOVA, which was followed by LSD or Tamhane tests. All analyses were carried out using SPSS 17.0 (Chicago, IL). A value of *P* < 0.05 was taken as a significant difference for all statistical analyses.

## 3. Results

### 3.1. Effect of RAS-RH on X-Ray-Induced Fibrosis

**Figure 1:**
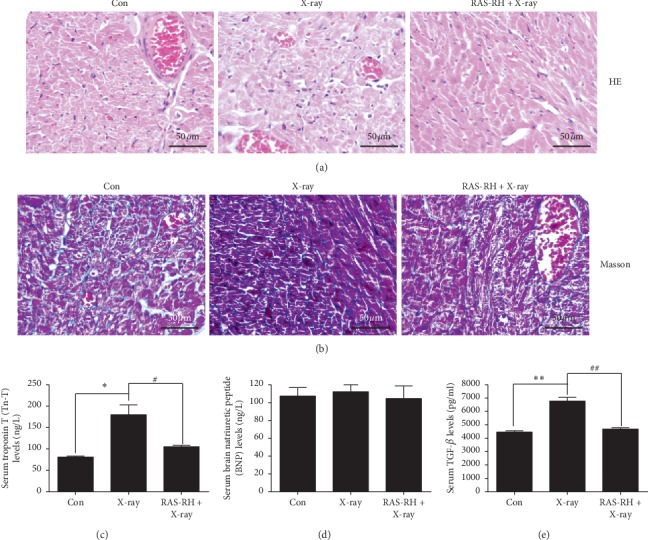
RAS-RH protects cardiac tissue from X-irradiation-induced injury and inflammation. (a) Representative images of hematoxylin & eosin staining for hearts from con, as well as X-ray and RAS-RH + X-ray groups mice after 30 days of RAS-RH treatment. (b) Representative images of Masson's trichrome staining of heart sections obtained from the three experimental groups. Blue staining indicates the deposition of collagen (400 × magnification). (c), (d), (e) Enzyme-linked immunosorbent assay (ELISA) analysis of TnT, serum brain natriuretic peptide (BNP), and TGF-*β*1 amount, respectively (*n* = 3, ^*∗*^*P* < 0.05,^*∗∗*^*P* < 0.01 vs. the control group; ^#^*P* < 0.05, ^##^*P* < 0.01 vs. the X-ray group).

Morphological changes in the heart from the three groups were examined by H&E staining and Masson's trichrome staining (Figures 1(a) and 1(b)). Cardiac histology from the control appeared morphologically normal, whereas in the X-ray radiation group, inflammatory infiltration was observed by H&E staining, and myocardial fibrosis and irregular collagen deposition in the hearts were shown by Masson's trichrome staining. The histology of heart tissue from the RAS-RH + X-ray group showed less inflammatory infiltration and myocardial fibrosis than what was observed in the X-ray radiation group. These results indicated that X-ray radiation can induce significant myocardial fibrosis, and 50 mg/kg/day of RAS-RH partially reduced X-ray-induced fibrosis.

To investigate the effect of RAS-RH on cardiac fibrosis under physiological measurements of cardiac function, serum levels of TnI and brain natriuretic peptide (BNP) were tested by ELISA (Figures 1(c) and 1(d)). TnT content from the X-ray radiation group (181.523 ± 23.407 ng/L) was significantly greater than that of the control group (81.906 ± 2.054 ng/L). After treatment with RAS-RH, there was significantly less TnT content in the RAS-RH + X-ray group (106.456 ± 3.106 ng/L) than there was in the X-ray radiation group (181.523 ± 23.407 ng/L). However, the levels of BNP were not elevated after X-irradiation. Moreover, after treatment with RAS-RH, there was no change in the expression level of BNP.

As active TGF-*β*1 plays a major role in X-irradiation-induced myocardial fibrosis, we next investigated serum TGF-*β*1 amount by ELISA, and as shown in Figure 1(e), TGF-*β*1 amount was significantly increased in the X-ray radiation group compared with that of the control group (6841.944 ± 283.360 pg/mL vs. 4504.815 ± 103.948 pg/mL, *P* < 0.01). Treatment with RAS-RH led to a 44.353% reduction in the TGF-*β*1 amount.

### 3.2. Effect of RAS-RH on X-Ray-Induced Expression of microRNA-200a, TGF-*β*1, smad3, and COL1*α*

**Figure 2:**
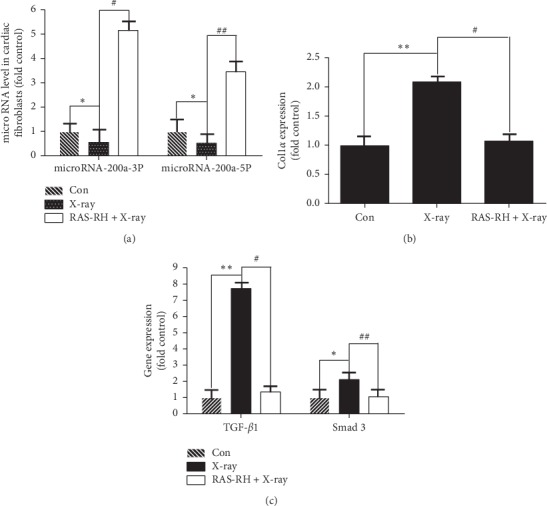
RAS-RH regulates the mRNA levels of microRNA-200a, COL1*α*, TGF-*β*1, and Smad3. Quantitative real-time RT-PCR analysis of microRNA-200a (a), COL1*α* (b), and TGF-*β*1 and Smad3 (c) mRNA levels in the Con, X-ray, and RAS-RH + X-ray groups (*n* = 3, ^*∗*^*P* < 0.05,^*∗∗*^*P* < 0.01 vs. the control group; ^#^*P* < 0.05, ^##^*P* < 0.01 vs. the X-ray group).

Next, we wanted to determine which pathways were involved in RAS-RH resistance to X-irradiation-induced fibrosis, so we performed quantitative real-time polymerase chain reactions and western blotting in primary rat cardiac fibroblasts to detect the mRNA and protein levels of microRNA-200a, TGF-*β*1, smad3, and COL1*α*. Our data revealed that microRNA-200a-3P and microRNA-200a-5P showed a similar expression pattern with a notable decrease after X-irradiation, which was followed by obvious upregulation after treatment with RAS-RH (Figure 2(a)). In addition, the expression of TGF-*β*1 and smad3 was significantly increased in the X-ray radiation treatment group compared with that of the control group. However, after treatment with RAS-RH, the expression level of TGF-*β*1 and smad3 mRNA was reduced 5.707-fold and 1.990-fold in comparison with that of the X-ray treatment group (Figure 2(c)). Furthermore, similar to TGF-*β*1 and smad3, COL1*α* gene expression was mainly elevated in the X-ray radiation group, whereas a decrease in COL1*α* expression level was observed in the RAS-RH + X-ray group (Figure 2(b)). These results indicate that microRNA-200a, TGF-*β*1, smad3, and COL1*α* are involved in RAS-RH resistance to X-irradiation-induced fibrosis.

**Figure 3:**
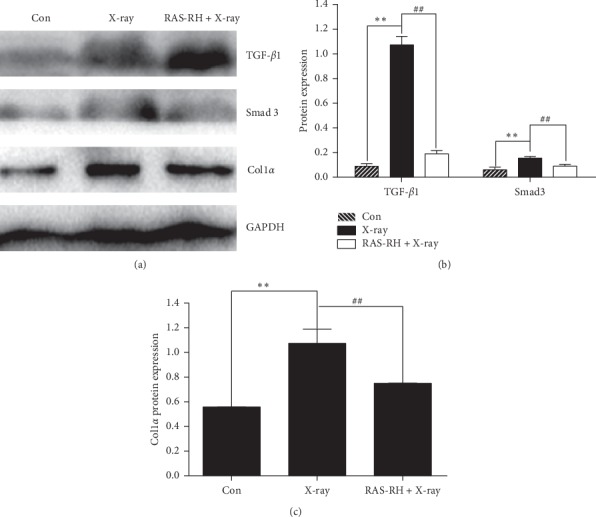
RAS-RH regulates the protein expression of TGF-*β*1, Smad3, and COL1*α*. (a) Western blotting images indicate the TGF-*β*1, Smad3, and COL1*α* protein expression in the Con, X-ray, and RAS-RH + X-ray groups. (b) Relative TGF-*β*1 and Smad3 protein expression levels were analyzed by Image-Pro Plus 6.0. (c) Relative COL1*α* protein expression levels were analyzed by Image-Pro Plus 6.0 (*n* = 3, ^*∗*^*P* < 0.05,^*∗∗*^*P* < 0.01 vs. the control group; ^#^*P* < 0.05, ^##^*P* < 0.01*P* < 0.01 vs. the X-ray group).

Consistent with the gene expression of TGF-*β*1, smad3, and COL1*α*, the protein expression of TGF-*β*1, smad3, and COL1*α* was severely elevated in the X-ray radiation group compared with that of the control group. However, after treatment with RAS-RH, the protein expression levels of TGF-*β*1, smad3, and COL1*α* were dramatically reduced compared with those of the X-ray treatment group (Figures 3(a)–3(c)).

### 3.3. Effect of RAS-RH on X-Ray-Induced Oxidative Stress

**Figure 4:**
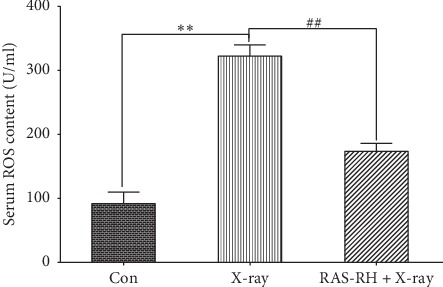
RAS-RH prevents X-irradiation-induced oxidative stress. ELISA analysis for serum ROS content in the Con, X-ray, and RAS-RH + X-ray groups (*n* = 3, ^*∗*^*P* < 0.05,^*∗∗*^*P* < 0.01 vs. the control group; ^#^*P* < 0.05, ^##^*P* < 0.01 vs. the X-ray group).

Because oxidative stress has been implicated in the pathogenesis of cardiac fibrosis, we assessed the changes in ROS formation in rat serum. As shown in Figure 4, compared to the control group, the serum ROS formation was strongly increased in the X-ray treatment group (*P* < 0.01). However, after treatment with RAS-RH, ROS formation in rat serum was reduced 1.855-fold in the RAS-RH + X-ray group compared with the X-ray treatment group.

### 3.4. Effect of RAS-RH on Apoptosis after X-Irradiation

**Figure 5:**
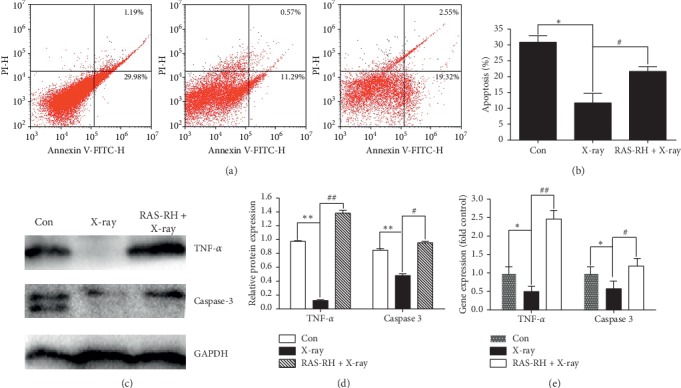
RAS-RH induces apoptosis in primary rat cardiac fibroblasts after X-irradiation. (a) Flow cytometry analysis of primary rat cardiac fibroblast apoptosis in the Con, X-ray, and RAS-RH + X-ray groups. (b) The bar graph shows a statistical analysis of image (a) results. (c) Western blotting images for TNF-*α* and caspase-3 protein expression in the Con, X-ray, and RAS-RH + X-ray groups. (d) Relative TNF-*α* and caspase-3 protein expression levels were analyzed by Image-Pro Plus 6.0. (e) Quantitative real-time RT-PCR analysis of TNF-*α* and caspase-3 mRNA levels (*n* = 3, ^*∗*^*P* < 0.05,^*∗∗*^*P* < 0.01 vs. the control group; ^#^*P* < 0.05, ^##^*P* < 0.01 vs. the X-ray group).

To investigate whether RAS-RH could induce apoptosis of primary rat cardiac fibroblasts after X-irradiation, we examined the percentage of apoptotic fibroblasts by flow cytometry. We found that the percentage of apoptotic fibroblasts was strongly decreased in the X-ray treatment group compared with that of the control group (*P* < 0.05). Conversely, we observed significantly increased apoptosis of fibroblasts after treatment with RAS-RH (Figures 5(a) and 5(b)). Our data suggest that fibroblast apoptosis may play an important role in RAS-RH mitigating X-irradiation-induced fibrosis. To determine which pathways are involved in RAS-RH-induced apoptosis after X-irradiation, we investigated the changes in apoptosis-related protein and gene expression in primary rat cardiac fibroblasts. We found that the mRNA and protein levels of apoptotic factors caspase-3 and TNF-*α* were significantly decreased in the X-ray group compared with those of the control group. After treatment with RAS-RH, caspase-3 and TNF-*α* mRNA and protein levels were strongly increased in the RAS-RH + X-ray group compared with those of the X-ray treatment group, suggesting that RAS-RH-induced apoptosis after X-irradiation may be partially related to caspase-3 and TNF-*α* (Figures 5(c)–5(e)).

## 4. Discussion

Our previous study indicated that the ultrafiltration extract of Danggui Buxue decoction protected cardiomyocytes against oxidative injury induced by hydrogen peroxide [16]. In addition, we also confirmed that RAS-RH has a correlative protective effect against RIMF [14]. Other studies have shown that Radix Angelica Sinensis and Radix Hedysari are effective in “the treatment of hepatic fibrosis and pulmonary fibrosis” [17]. Moreover, Radix Hedysari seemed to have a greater antifibrosis effect and greater immunological bioactivity than Astragali Radix in organ tissue [18]. We prepared an ultrafiltration extract of Radix Angelica Sinensis and Radix Hedysari (RAS-RH) according to the instruction of the manufacturer of the Danggui Buxue decoction. The RAS-RH exerted antifibrosis activity in rats with RIMF based on the following evidence. (a) RAS-RH ameliorated X-ray induced collagen deposition, which was observed on the morphological level by Masson staining in the rat heart; collagen deposition predicts the occurrence of fibrosis. (b) RAS-RH reduced the expression of troponin *T* in rat hearts exposed to X-ray radiation, and troponin *T* expression is a typical phenotype of cardiomyocyte damage. We also confirmed that RAS-RH regressed circulating amounts of TGF-*β*1 in rat serum. The elevated levels of collagen, troponin *T*, and TGF-*β*1 in the rat have a positive relationship with the degree of RIMF, so RAS-RH protects against X-ray-induced fibrosis in a rat heart fibrosis model.

We successfully established a rat model of X-ray radiation-induced myocardial fibrosis and then explored potential mechanisms of fibrosis development. RIMF is characterized by the occurrence of diffuse interstitial fibrosis [2], the presence of cardiac fibroblasts that play a vital role in secreting TGF-*β* and collagen [3], and the amount of TGF-*β*1, which triggers the downstream Smad3 signaling pathway [19]. Amplified TGF-*β*1/Smad3 signaling cascades in fibroblasts accelerate the progression of fibrosis induced by X-ray exposure and result in the expression of COL1*α*. Meanwhile, microRNA-200a is involved in the occurrence of RIMF. Circulating microRNA-200a may represent a novel marker in patients with hypertrophic cardiomyopathy [7]. After RT, microRNA-200a is significantly downregulated in patients with lung cancer [20], while the expression of microRNA-200a is higher in patients with head and neck squamous cell carcinoma 12 months after RT [21]. Our results demonstrate that TGF-*β*1, Smad3, and COL1*α* are significantly increased in X-ray-induced cardiac fibroblasts compared with those of controls. After RAS-RH treatment, the opposite trend subsequently appeared. Contrary to the above trend, the expression of microRNA-200a was obviously reduced in cardiac fibroblasts 1 month after X-ray radiation, and RAS-RH markedly elevated X-ray radiation-induced decreases in microRNA-200a. Moreover, microRNA-200a-3P and microRNA-200a-5P are involved in RIMF. A previous study reported that there was negative regulation between TGF-*β*1/Smad3 and microRNA-200a [22], which is consistent with our results and indicates that the dysregulation of TGF-*β*1/Smad3-mediated microRNA-200a is a potential pathophysiological mechanism of RIMF. RAS-RH treatment appears to ameliorate X-ray-induced myocardial fibrosis via downregulation of TGF-*β*1, Smad3, and COL1*α* and via upregulation of microRNA-200a.

A heart exposed to X-ray radiation generates large quantities of ROS, which are responsible for oxygen stress damage [6]. ROS production induced by radiation disturbs normal molecular mechanisms and stimulates fibroblasts to secrete TGF-*β*1. Feed-forward loops, which activate ROS, amplify profibrotic cascaded signals that induce myofibroblast accumulation and collagen disposition in RIMF [23]. Our study demonstrates that X-ray radiation leads to an increase in ROS formation and that RAS-RH treatment inhibits this increase. These data suggest that oxygen stress is the main pathological factor in RIMF and that there is a positive relationship between TGF-*β*1/Smad3 and ROS.

An increase in fibroblastic resistance to apoptosis is associated with RIMF, and apoptosis resistance in fibroblasts leads to progressive fibrosis [24]. Fibroblasts exposed to radiation have increased protection against oxidative stress damage and decreased sensitivity to apoptosis [25]. TNF-*α* incites cardiac fibroblast apoptosis and provokes adverse cardiac remodeling [26]; moreover, TNF-*α* triggers cardiac fibroblast adhesion and aggravates myocardial fibrosis [27]. Radiation selectively activates caspase-3 expression, which mediates a proapoptotic signaling pathway [28]. Thus, the role of TNF-*α* and caspase-3 in cardiac fibroblast exposure to X-ray exposure is vital. In this study, X-ray radiation reduced cardiac fibroblast apoptosis, and the expression of TNF-*α* and caspase-3 was lower in cardiac fibroblast exposed to X-ray radiation than it was in the control. After RAS-RH treatment, the protein and mRNA levels of TNF-*α* and caspase-3 increased compared to those in the X-ray group, which implies that apoptosis is mechanistically involved in RIMF; however, when apoptosis resistance may occur in cardiac fibroblast exposure to X-ray, the sensitivity to apoptosis molecules of TNF-*α* and caspase-3 in cardiac fibroblasts decreases, so RAS-RH treatment may ameliorate apoptosis resistance and promote fibroblast apoptosis.

## 5. Conclusion

Based on our above study and previous reports, the occurrence of RIMF is highly related to microRNA-200a, ROS, TGF-*β*1/Smad3, and cardiac fibroblast apoptosis. We propose the following mechanism: the effect of RAS-RH mitigates X-ray-induced cardiac fibrosis in rats exposed to X-ray radiation. The X-ray radiation leads to an immediate increase in both ROS and TGF-*β*1/Smad3 and a decrease in microRNA-200a that partially stimulates the production of ROS and the activation of TGF-*β*1/Smad3, and it promotes the secretion of TGF-*β*1 from cardiac fibroblasts. Feed-forward loops between ROS and TGF-*β*1/Smad3 amplify profibrotic signals, which promote fibroblast proliferation and ultimately fibrosis. RAS-RH exerts a protective effect against X-ray-induced myocardial fibrosis.

## Figures and Tables

**Table 1:** Primer sequences used for real-time RT-PCR analyses.

Gene	Corporation	Catalog#
microRNA-200a-3P	FulenGen	RmiRQP0298
microRNA-200a-5P	FulenGen	RmiRQP3166
U6	FulenGen	RmiRQP9003
Col1*α*	FulenGen	RQP054226
TGF-*β*1	FulenGen	RQP045788
Smad3	FulenGen	RQP090103
ROS	FulenGen	RQP084253
TNF-*α*	FulenGen	RQP089950
Caspase-3	FulenGen	RQP049241
GAPDH	FulenGen	RQP049537

## Data Availability

The data used to support the findings of this study are included within the article.
